# A Novel Regulator Couples Sporogenesis and Trehalose Biogenesis in *Aspergillus nidulans*

**DOI:** 10.1371/journal.pone.0000970

**Published:** 2007-10-03

**Authors:** Min Ni, Jae-Hyuk Yu

**Affiliations:** 1 Department of Bacteriology, University of Wisconsin, Madison, Wisconsin, United States of America; 2 Department of Genetics, University of Wisconsin, Madison, Wisconsin, United States of America; Institut Pasteur, France

## Abstract

Trehalose is a compatible osmolyte produced by bacteria, fungi, insects and plants to protect the integrity of cells against various environmental stresses. Spores, the reproductive, survival and infection bodies of fungi require high amounts of trehalose for long-term survival. Here, via a gain-of-function genetic screen, we identify the novel regulator VosA that couples the formation of spores and focal trehalose biogenesis in the model fungus *Aspergillus nidulans*. The *vosA* gene is expressed specifically during the formation of both sexual and asexual spores (conidia). Levels of *vosA* mRNA and protein are high in both types of spore. The deletion of *vosA* results in the lack of trehalose in spores, a rapid loss of the cytoplasm, organelles and viability of spores, and a dramatic reduction in tolerance of conidia to heat and oxidative stress. Moreover, the absence of *vosA* causes uncontrolled activation of asexual development, whereas the enhanced expression of *vosA* blocks sporulation, suggesting that VosA also functions in negative-feedback regulation of sporogenesis. VosA localizes in the nucleus of mature conidia and its C-terminal region contains a potential transcription activation domain, indicating that it may function as a transcription factor primarily controlling the late process of sporulation including trehalose biogenesis. VosA is conserved in most fungi and may define a new fungus-specific transcription factor family.

## Introduction

Organisms have evolved various adaptive mechanisms to survive unfavorable environmental conditions. Trehalose, found in a wide variety of organisms, is a well-suited osmolyte and superior stabilizer of proteins and membranes. This specialized sugar protects cellular integrity and functions from various stresses including dehydration, heat, cold, and oxidation [1], [2].

Fungi produce spores as the main means of propagation, survival and infection. Fungal spores are found in every environment inhabited by humankind and have a significant impact on everyday life [3], [4]. These spores contain large quantities of trehalose [1], which is necessary for long-term viability. However, the molecular mechanism coupling trehalose biogenesis and the formation of spores is unknown.

The genus *Aspergillus* includes the most common fungi having both beneficial and detrimental impacts on human activities. All species reproduce asexually by forming long chains of asexual spores (conidia) radiating from a specialized structure called a conidiophore. Among them, *Aspergillus nidulans* has served as an excellent model for studying the mechanisms of asexual development (conidiation) [5], [6].

A key step for conidiation is the activation of *brlA*, which encodes a C_2_H_2_ zinc finger transcription factor (TF) [7]. Further studies identified and characterized *abaA* and *wetA* as also being important in this process. The *abaA* gene encodes a potential TF that is activated by *brlA* during the middle stages of conidiation [8]. WetA functions in the late phase of development and activates genes involved in the synthesis of spore wall components [9], [10]. These three genes have been proposed to define a central regulatory pathway that controls ordered expression of conidiation-specific genes during conidiophore development and spore maturation [5], [6], [11]. Notably, later studies have revealed that BrlA is required for conidiation in two other aspergilli, the opportunistic human pathogen *A. fumigatus* [12] and the industrial fungus *A. oryzae* [13], indicating that its function is conserved in the genus *Aspergillus*.

However, a simple model depicting the linear activation of *brlA*→*abaA*→*wetA* is incomplete and unable to explain tight regulation of these genes. For instance, transcripts of *brlA* and *abaA* accumulate at the early/middle phases, diminish during the late phase of conidiation and are undetectable in mature conidia [5], [14]. On the contrary, *wetA* mRNA level reaches the maximum during the late phase of conidiation and remains high in mature conidia [5], [9]. These suggest that another regulator may coordinate the expression of conidiation-specific genes.

In this study, through a gain-of-function genetic screen, we identify the novel regulator VosA that couples the completion of sporogenesis and focal accumulation of trehalose in spores. VosA is highly conserved in most (if not all) filamentous and dimorphic fungi and is similar to three other proteins including VeA, a well-known regulator of fungal development and secondary metabolism [15]–[18]. VosA is required for the biogenesis of trehalose during spore formation, thereby for the long-term survival of both conidia and sexual spores (ascospores). As it functions as a key negative-feedback regulator of conidiation, *vosA* is necessary and sufficient to direct repression of *brlA* and development. We further demonstrate that VosA is a potential TF and localizes in the nucleus of mature conidia, and propose that the conserved VosA/VeA (or *velvet*) class proteins may define a new fungal TF family.

## Results

### Identification of *vosA*

**Figure 1:**
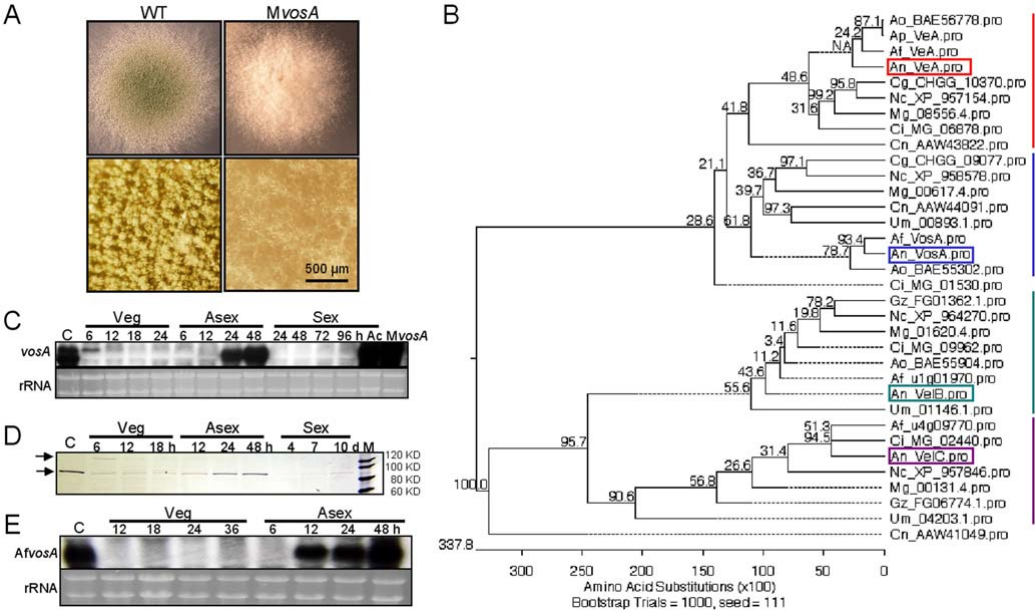
Synopsis of *vosA*. (A) Colonies of WT (RNIW5) and multi-copy *vosA* (M*vosA*) strains grown on solid MM at 37°C for 3 d together with the close-up views (lower panel). (B) A phylogenetic tree of proteins similar to VosA generated by MegAlign in Lasergene v7.0 (DNASTAR). ClustalV method was used for protein alignment. An: *A. nidulans*, Af: *A. fumigatus*, Ao: *A. oryzae*, Ap: *A. parasiticus*, Ci: *Coccidioides immitis*, Mg: *Magnaporthe grisea*; Cg: *Chaetomium globosum*; Nc: *Neurospora crassa*; Um: *Ustilago maydis*; Cn: *Cryptococcus neoformans*; Gz: *Gibberella zeae*. (C) Levels of *vosA* mRNA throughout the lifecycle of WT. Numbers indicate the time (h) of incubation in liquid MM (Veg) and post-asexual (Asex) or sexual (Sex) developmental induction. C and Ac represent conidia and ascospores. Last lane shows elevated *vosA* mRNA level in the M*vosA* colony grown on solid MM for 3 d. (D) Levels of the VosA protein throughout the lifecycle of a *vosA*(p)::VosA::FLAG strain (TNI10.34.1). Arrows indicate two protein bands. (E) Levels of Af*vosA* mRNA during the lifecycle of *A. fumigatus* WT.

We hypothesized that a gain-of-function (multi-copy) genetic screen would identify novel negative regulators of development that cannot be defined via chemical mutagenesis. Briefly, a wild type (WT) strain was transformed with the pRG3-AMA1 based WT library [19] and out of more than 50,000 transformants seven exhibiting non-sporulating phenotypes were isolated. By direct sequencing of the insert ends of plasmids recovered from these transformants followed by a genome search [20], three multi-copy repressors of development have been identified: AN6437 and AN1959 from one transformant each, and AN6578 from four transformants. The plasmid from one transformant remains to be identified. We then found that reintroduction of the plasmid containing the AN1959 locus alone resulted in a complete loss of development (Fig 1A). The AN1959 gene is further studied and found to be essential for the **v**iability **o**f **s**pores, thus named as ***vosA*** (see below).

The sequence analyses of the RT-PCR products further led to the identification of the *vosA* ORF composed of 1950 bp with 10 exons predicted to encode a 430 aa-length protein (DQ856465). VosA is similar to three other proteins including VeA [15], VelB (EF540815) and VelC (EF540816) in *A. nidulans*. These *velvet* family proteins are highly conserved in both ascomycetes and basidiomycetes (Fig 1B).

Levels of the VosA transcripts and protein(s) are high in conidia (C), ascospores (Ac) and during the late phase of conidiation when conidia differentiate and become mature (Fig 1C&D). During vegetative growth, levels of the *vosA* transcripts and protein(s) quickly drop and continue to be undetectable (or low) until 24 h post induction of conidiation. The *vosA* gene appears to consist of two overlapping transcripts (1.8 kb and 2.4 kb), where only the 2.4 kb transcript is present in the early phase (6 h) of vegetative growth. Two protein bands (90 KDa and 120 KDa) are also detectable at this time. The *A. fumigatus* VosA homolog (AfVosA; EF544392) shows 79% aa identity and exhibits the almost identical mRNA accumulation pattern (Fig 1E), implying that VosA function might be conserved in aspergilli.

### VosA is required for the viability of spores

**Figure 2:**
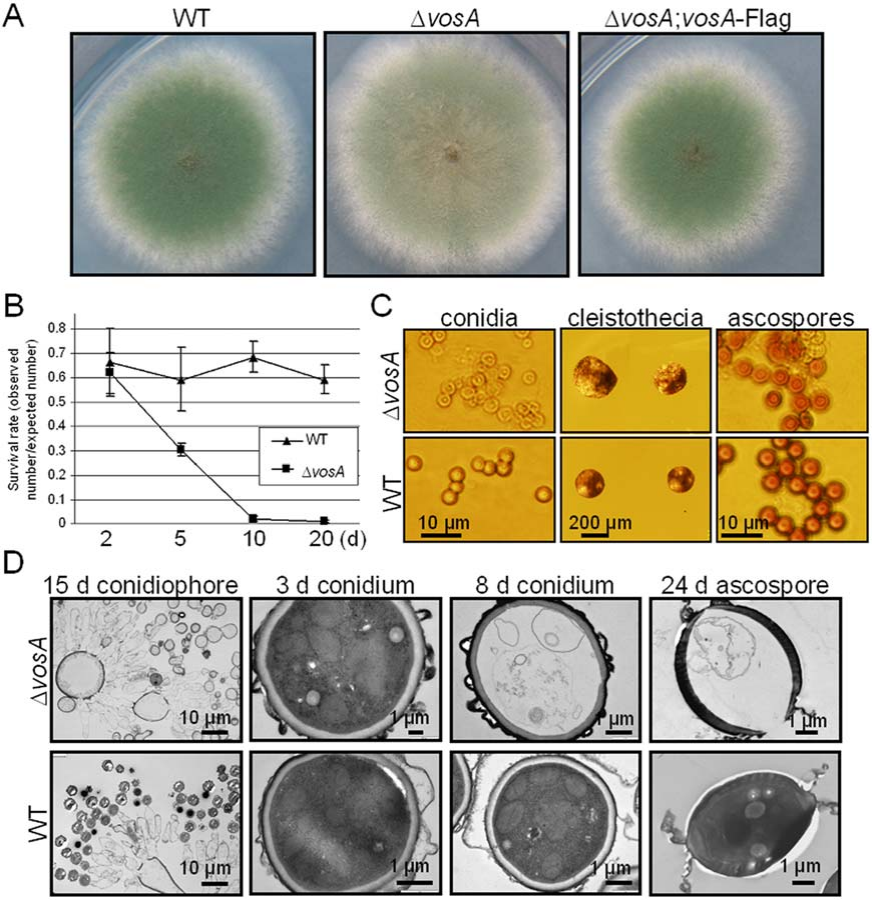
Phenotypes resulting from Δ*vosA*. (A) Photographs of the colonies of WT (FGSC26), Δ*vosA* (RNI10.2) and complemented (Δ*vosA*+*vosA*-FLAG; TNI10.34.1) strains grown on solid MM for 3 d. (B) Viability of the conidia of WT and Δ*vosA* strains grown at 37°C for 2, 5, 10 and 20 days. (C) Photomicrographs of 20 d old conidia, 3 month old cleistothecia and ascospores of Δ*vosA* and WT strains. (D) TEM images of 15 d old conidiophores, 3 d and 8 d old conidia, and 24 d old ascospores of Δ*vosA* and WT strains.

The *vosA* deletion (Δ*vosA*) mutant produces light green conidia that differ from WT (Fig 2A). Noticeably, Δ*vosA* causes a radical decrease in the viability of spores. When conidia of 2, 5, 10 and 20 d grown colonies of WT and Δ*vosA* strains are compared, the mutant conidia quickly lose viability starting from 5 d (Fig 2B) and become translucent (Fig 2C). Furthermore, the Δ*vosA* mutant produces defective sexual fruiting bodies (cleistothecia) containing few (∼1%) viable semi-transparent ascospores (Fig 2C).

To understand the cellular nature of the defects, conidia of WT and Δ*vosA* strains were examined by Transmission Electron Microscopy (TEM) and Scanning Electron Microscopy (SEM). While no clear differences were observed in SEM (not shown), TEM has revealed that an electron-light layer found in the WT spore wall is absent in the Δ*vosA* mutant spores. Moreover, both Δ*vosA* conidia and ascospores appear to lack cytoplasm and organelles including the nucleus (Fig 2D). In addition, no genomic DNA or RNA was detectable in 8 d or older mutant conidia (not shown). These results indicate that VosA is essential for the integrity of both asexual and sexual spores.

### VosA is essential for trehalose accumulation in conidia

**Figure 3:**
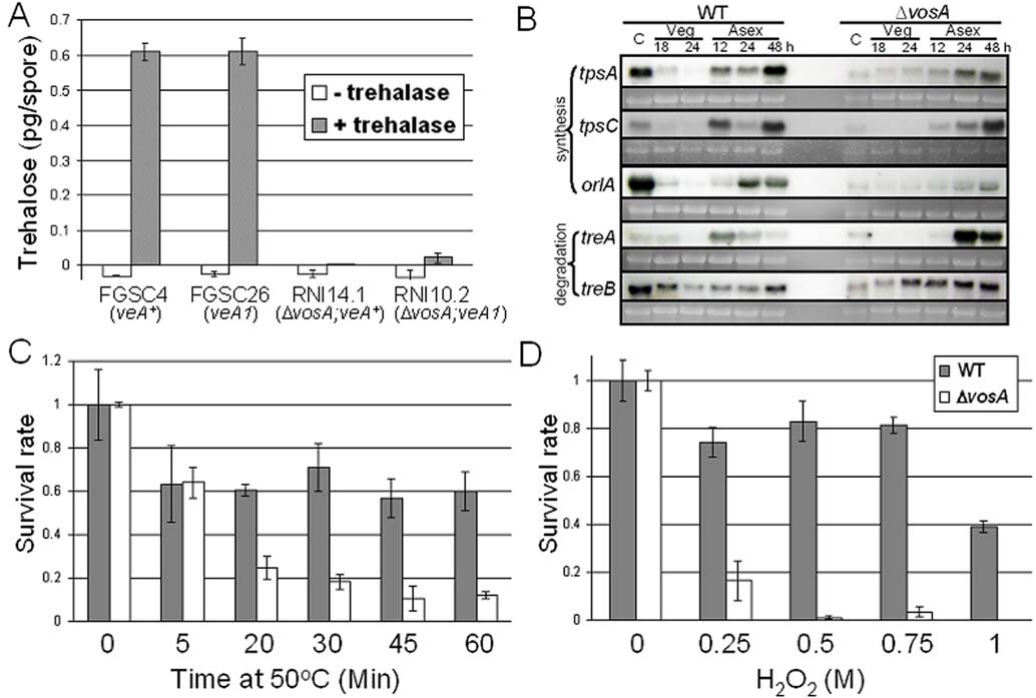
Requirement of VosA for trehalose accumulation and stress tolerance. (A) The amount of trehalose (pg) per conidium in the freshly collected 2 d old conidia of WT (FGSC4, *veA* ^+^ and FGSC26, *veA1*) and Δ*vosA* (RNI14.1, Δ*vosA*; *veA* ^+^ and RNI10.2, Δ*vosA*; *veA1*) strains (triplicate). No trehalase treatment served as a negative control. (B) Levels of *tpsA*, *tpsC*, *orlA*, *treA* and *treB* transcripts in WT (FGSC26) and Δ*vosA* (RNI10.2) strains. (C–D) Tolerance of WT (filled bar) and the Δ*vosA* mutant (open bar) to heat (C) or oxidative (D) stress.

The deletion of the *A. nidulans tpsA* gene encoding trehalose-6-phosphate (T-6-P) synthase causes a rapid loss of spore viability [21]. Because no apparent differences in the spore wall composition between the WT and Δ*vosA* conidia were detectable, we asked whether VosA is needed for the proper accumulation of trehalose in spores. Measurement of trehalose amount in 2 d old conidia of WT and Δ*vosA* strains reveal that trehalose is undetectable in the Δ*vosA* conidia (Fig 3A). This lack of trehalose was observed regardless of the presence (*veA^+^*) or absence (*veA1*) of the fully functional VeA protein, indicating that VosA's function in trehalose biogenesis is independent of VeA.

To investigate the molecular basis of the defects, we examined the mRNA levels of selected genes associated with synthesis (*tpsA*, *tpsC* and *orlA*) or breakdown (*treA* and *treB*) of trehalose [21]–[24, Christophe d'Enfert, personal communication]. The deletion of *vosA* results in reduced *tpsA*, *tpsC* and *orlA* mRNA levels during asexual development and in mature conidia (Fig 3B). On the contrary, mRNA levels of *treA* (but not *treB*) increase during the formation and maturation of conidia. These suggest that VosA is required for proper regulation of genes necessary for the accumulation of trehalose in spores. Supporting the positive regulatory role of VosA in trehalose biogenesis, overexpression of *vosA* is sufficient to direct upregulation of *tpsA*, *tpsC* and *orlA* even in vegetative cells (Fig S1). We also tested whether the Δ*vosA* mutant conidia show altered stress responses and found that the mutant conidia exhibit a drastic reduction in tolerance to heat (Fig 3C) and H_2_O_2_ (Fig 3D), corroborating the idea that trehalose functions as a key protectant. The viability of the Δ*vosA* mutant was not affected by increased osmolarity in the medium or storage solution (data not shown).

### VosA regulates conidiation-specific gene expression

**Figure 4:**
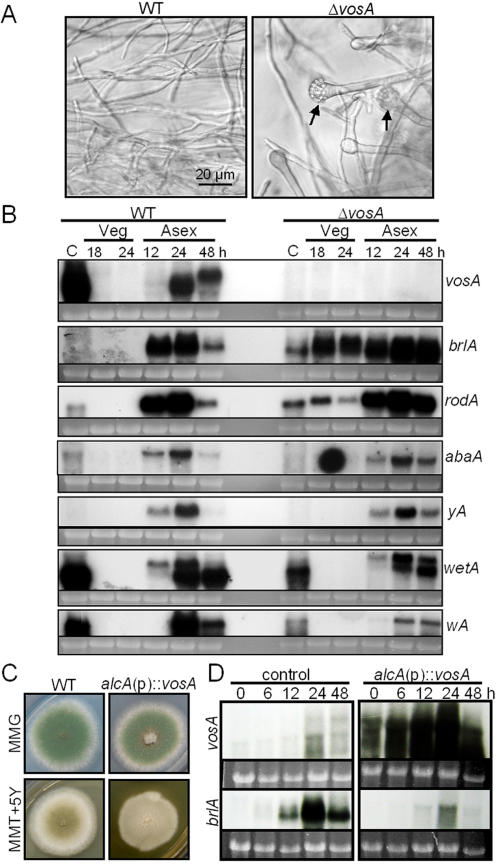
VosA is a negative regulator of development. (A) Hyperactive conidiation caused by Δ*vosA* (photographed at 24 h in liquid MM). The arrows indicate conidiophores. (B) Levels of *vosA*, *brlA*, *rodA*, *abaA*, *yA*, *wetA* and *wA* transcripts in WT (FGSC26) and Δ*vosA* (RNI10.2) strains in liquid MM (Veg) and post developmental induction (Asex). A dot between the Δ*vosA* Veg 18 and 24 lanes in *abaA* hybridization is an artifact. (C) Photographs of the colonies of WT (FGSC26) and *vosA* overexpression (TNI9.1) strains grown on non-inducing (MMG) and inducing (MMT + YE) medium at 37°C for 3 d. (D) Northern blot for levels of *vosA* and *brlA* mRNA in control (TJA53.1) and *alcA*(p)::*vosA* (TNI9.1) strains.

As VosA plays a key role in modulating conidiation, Δ*vosA* results in the formation of conidiophores in liquid submerged culture (arrows in Fig 4A) where WT strains do not develop conidia. Moreover, M*vosA* blocks conidiation completely even under the conditions favoring development (Fig 1A). To further verify the repressive role of *vosA* in conidiation, we generated the *vosA* overexpression mutant by fusing the *vosA* ORF with the inducible *alcA* promoter [25]. As elevated expression of *vosA* is sufficient to block development, overexpression of *vosA* inhibits conidiation (Fig 4C). We also found that the VosA-mediated inhibition of development is proportional to the dosage of *vosA*, and that three copies of *vosA* are sufficient to block development (not shown).

To further investigate the regulatory role of *vosA* in conidiation, we examined the mRNA levels of various development-specific genes. As shown in Figure 4B, Δ*vosA* results in nearly constitutive accumulation of high levels of *brlA* (and *rodA* [26]) mRNA even at 48 h post developmental induction and in conidia. Conversely, levels of *wetA* (and *wA* [27]) mRNA decrease considerably in the Δ*vosA* mutant (note the differences in conidia). Moreover, consistent with the phenotype, overexpression of *vosA* causes the blockage in accumulation of *brlA* mRNA (Fig 4D). Collectively, these results indicate that VosA inhibits *brlA* expression, but may activate *wetA*. In addition, Δ*vosA* causes altered expression of *yA* encoding a conidial laccase [28] and *wA* encoding a polyketide synthase [27], likely leading to lighter conidial pigmentation.

To further dissect genetic interactions among the four regulators, levels of *vosA* mRNA were examined in various mutants including *brlA42*, *abaA14*, *wetA6*, *alcA*(p)::*brlA*, *alcA*(p)::*brlA abaA14* and *alcA*(p)::*abaA* (Fig S2 and S3). Expression analyses reveal that both *abaA* and *wetA* are required for *vosA* mRNA accumulation, and *vosA* and *wetA* activate each other. In addition, *wetA* is necessary for the repression of both *brlA* and *abaA* (see Discussion).

### VosA is a potential TF and localizes in the nucleus of mature conidia

**Figure 5:**
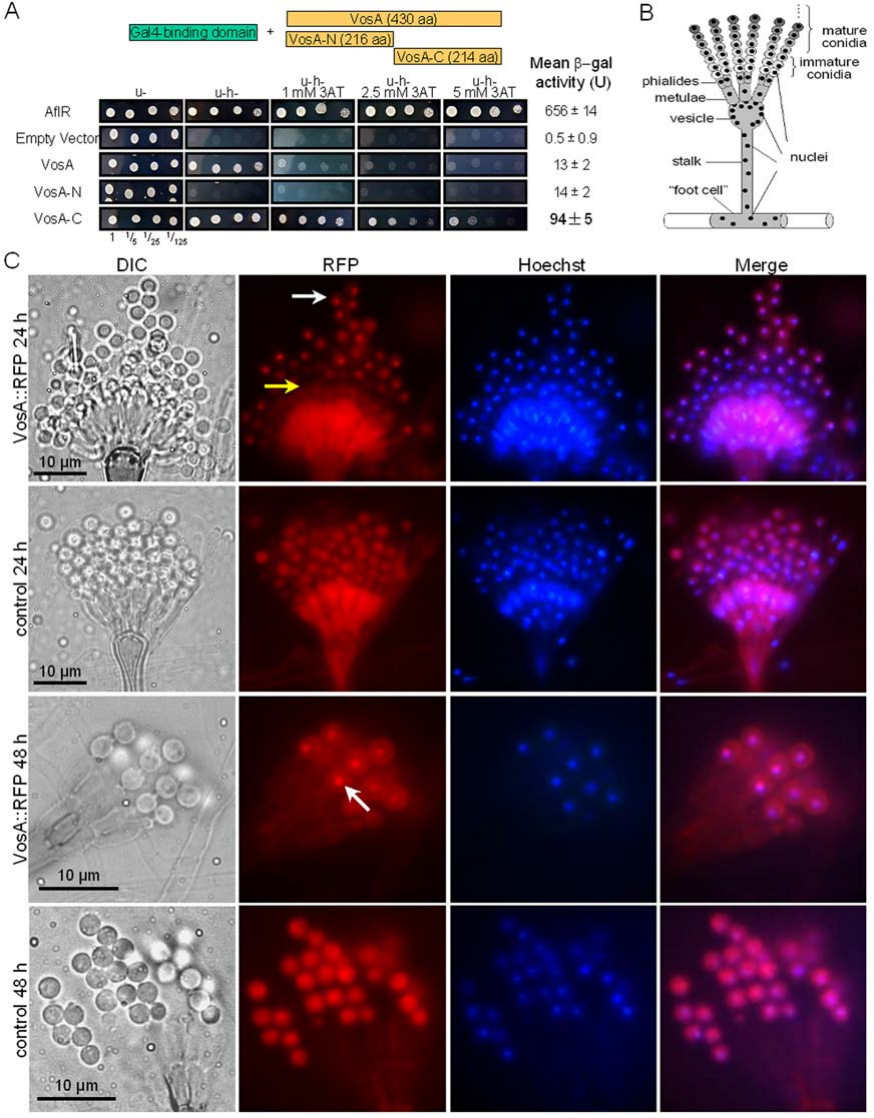
VosA is a potential TF and localizes in the nucleus of mature conidia. (A) Designated transformants were spotted in serial dilutions on Ura- MM (u-) and Ura-His- MM (u-h-) with various concentrations of 3AT. Yeast colonies were photographed after incubation at 30°C for 48 h. Mean LacZ activity (+/−SD; triplicate) is shown. (B) A simplified diagram of a conidiophore. (C) Control (*gpdA*[p]::RFP; TNI20.1) and *vosA*(p)::VosA::RFP (TNI13.3) strains were incubated on solid MM at 37°C for 24 and 48 h. Conidiophores were fixed and stained with Hoechst 33258 and images of DIC, RFP, Hoechst and the merge of RFP and Hoechst are shown. Yellow arrow indicates newly formed conidia and white arrow indicates mature conidia. Note that VosA mainly localizes in the nucleus of mature conidia.

Although no known DNA-binding domain is identified in its sequence, VosA contains a nuclear localization signal (NLS)-pat7 (241PVKRQRT247) and has a 65.2% likelihood of being localized in the nucleus as predicted by PSORT II (http://psort.nibb.ac.jp). We carried out the yeast-one hybrid assay by fusing the Gal4 DNA binding domain with full-length (VosA), N-terminal half (VosA-N) or C-terminal half (VosA-C) of VosA as well as AflR, a Gal4 type TF in *A. nidulans* [29], [30]. The chimeric proteins were expressed in yeast under the control of the *ADH1* promoter [31]. The transformants expressing VosA and VosA-C grow on the medium lacking histidine in the presence of 3AT as high as 1 mM and 5 mM, respectively (Fig 5A). Moreover, the strain expressing VosA-C shows six times higher β-galactosidase activity than those expressing VosA and VosA-N, implying that the C-terminal region of VosA contains a potential transcriptional activation domain.

We then examined the expression and localization of VosA during conidiophore development (see Fig 5B for structure). At 24 h, when conidia are actively differentiating, VosA-RFP accumulates in the cytoplasm of metulae, phialides and newly generated conidia (yellow arrow), and it begins to localize in the nucleus of mature conidia (white arrow in Fig 5C). A strain expressing RFP under the control of the *gpdA* promoter shows even distribution of RFP in the cytoplasm of all cell types. At 48 h, when all conidia become mature, VosA-RFP localizes mainly in the nucleus of mature conidia (Fig 5C). These imply that the VosA protein is actively expressed in metulae and phialides, and localizes primarily in the nucleus of mature conidia.

## Discussion

In this study, we present the data showing that the multifunctional regulator VosA plays an essential role in long-term survival of both asexual and sexual spores likely via coupling sporogenesis and trehalose biogenesis. We have focused on dissecting the roles of VosA in *A. nidulans* asexual lifecycle.

**Figure 6:**
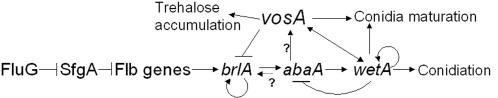
Model for regulation of conidiation (see text).

A conidiophore is composed of a thick-walled foot cell, a stalk, a multinucleate vesicle, two layers of uninucleate cells (metulae and phialides) and a large number of conidia (Fig 5B; [6], [32]). After being generated from phialides through repeated asymmetric mitotic cell divisions, conidia need to undergo a maturation process to enable the long-term viability [9]. Two key events in this process are the formation of a remarkably rigid four-layered conidial wall [9] and accumulation of trehalose [23], [33]. We demonstrate that maturation of spores requires VosA, which triggers an active buildup of trehalose and assists in the proper formation of the conidial wall. Importantly, we also found that the deletion of the *vosA* homolog in *A. fumigatus* results in reduced trehalose amount (∼50% of WT) in conidia and decreased viability (data not shown), suggesting that VosA function may be conserved in other aspergilli.

During the development of conidiophores, the expression and localization of central regulatory components *brlA*, *abaA* and *wetA* is tightly controlled [7]–[9], [14], [34]. We have presented a series of data supporting the crucial role that VosA plays in the balanced expression of these and other genes. VosA potentially functions as both an activator (for *wetA*, *tpsA* and *orlA*) to complete sporogenesis and a repressor (for *brlA* and *treA*) to confer negative-feedback regulation. Notably, *vosA* itself is subject to strict spatiotemporal regulation: 1) high levels of the VosA protein are first found in the cytoplasm of metulae, phialides and newly generated conidia, and then in the nucleus of mature conidia, and 2) the levels of the *vosA* mRNA and protein decrease rapidly during conidial germination and vegetative growth, which presumably allows another round of development to occur. However, it is important to note that the VosA protein (90 kDa) is present at low levels during the vegetative growth phases of WT (Fig 1D), and that Δ*vosA* results in extremely high levels of *brlA* mRNA accumulating in liquid culture conditions (Fig 4B). These suggest that VosA may maintain its repressive role on *brlA* even in vegetative cells to inhibit precocious development. Taken together, we propose that VosA and SfgA, an upstream repressor of conidiation with a Zn(II)_2_Cys_6_ domain [35], [36], confer differential modulation of conidiation and both are needed for proper control of asexual lifecycle.

A new genetic model depicting the regulation of conidiation is presented (Fig 6). In this model, during the early phases of growth, SfgA (and VosA) stay active thereby repressing the expression of the Flb and *brlA* genes [36]. The main function of the early developmental activator FluG is to remove the repressive effects imposed by SfgA [36]. This de-repression initiates the subsequent activation of Flbs (encoding TFs) and the central regulatory pathway. In conjunction with the differentiation of conidia, *vosA* is activated, which in turn represses *brlA* expression and confers maturation of conidia. As the simplest model, the repressive roles of *abaA* and *wetA* on the expression of *brlA* may be primarily attributed to the VosA functioning in a negative-feedback loop. The observations that both *brlA* and *wetA* are subject to auto-activation [5], [6] and that *wetA* functions in negative regulation of *abaA* (Fig S3) are indicated.

While our studies have revealed the important functions of VosA in development, much remains to be learned. The first task is to further investigate the molecular mechanisms for VosA-mediated regulation of sporogenesis and trehalose biosynthesis through identification of its potential targets by genome-wide screening and/or targeted approaches. For instance, examining the ability of VosA to bind the promoters of *brlA*, *wetA*, *tpsA* and *orlA* should help to test the postulated regulatory role of VosA (Fig 6). The second challenge is to characterize the functions of VosA homologs in other fungi. For instance, trehalose is required for plant infection by the rice pathogen *M. grisea* [37], in which a VosA homolog is found. It is of interest to see if the VosA homolog functions in various pathogenic fungi similarly. Thirdly, identification of VosA interacting proteins (if any) would be crucial to understand the mechanistic nature of VosA-mediated developmental regulation.

Our preliminary data indicate that VeA and VelB may also contain a transcriptional activation domain (not shown). Together with a set of evidence for the likelihood of VosA being a TF, it can be proposed that these *velvet* family proteins may define a new fungus-specific TF class. Moreover, given the pleiotropic effects caused by the mutational inactivation of VosA and VeA [15]–[18], these regulators may act globally at a higher hierarchic level influencing multiple biological processes. Additional studies of the *velvet* family proteins will further illuminate the molecular mechanisms interconnecting fungal morphogenesis and metabolism.

## Materials and Methods

### Fungal strains and growth

*Aspergillus* strains are listed in supporting Table S1. Standard culture and genetic techniques were used [38]. Strains were grown on minimal solid or liquid medium with appropriate supplements (simplified as MM; [39]). Induction of asexual or sexual development was done as described [35].

### Cloning of *vosA*

The recipient strain RNIW5 (*pyrG89*; *pyroA4*) was transformed with the pRG3-AMA1-*Not*I WT library [19] and four transformants showing a non-sporulating phenotype were isolated. The plasmids were recovered as described [36]. Direct sequencing of the insert ends identified the *vosA* gene (locus *AN1959.2*). The *vosA* ORF was determined by RT-PCR followed by sequence analyses.

### Plasmids

The *alcA*(p)::*vosA* construct was created as described [40] and cloned into the *Bam*HI site of pJW53 (JW Bok and NP Keller, unpublished) resulting in pNI16. The *vosA*(p)::VosA::RFP (mRFP1; [41]) construct was cloned into the *Bam*HI site of pSH96 [42] to generate pNI18. The RFP control construct was generated by fusing RFP with the *gpdA* promoter [43] and cloned into the *Eco*RI site of pJW53 giving rise to pNI21. For the yeast-one hybrid assay, cDNA of the coding regions of *aflR*, *vosA*, *vosA*-N (N-terminal 1∼216^th^ aa) or *vosA*-C (C-terminal 217∼430^th^ aa) was amplified via RT-PCR and cloned between the *Eco*RI and *Bam*HI (for *aflR*) or *Bam*HI and *Cla*I (for *vosA*, *vosA*-N and *vosA*-C) sites in pGBDUC1 [31] resulting in pNI32, 33, 34 and 35, respectively.

### Construction of *Aspergillus* strains

The *vosA* deletion mutant TNI2.1 was generated by transforming JAS26 with the *vosA* deletion construct with *argB* ^+^ [40]. The *A. fumigatus vosA* deletion mutants (TNI17.1-3) were generated by transforming AF293.1 with the Af*vosA* deletion construct containing Af*pyrG* ^+^. RNI10.2 (Δ*vosA*; *veA1*) was isolated from the cross between PW1 and TNI2.1. RNI14.1 (Δ*vosA*; *veA^+^*) was isolated from the cross between RRAW16 and RNI10.2. The plasmids pNI16, pNI18 and pNI21 were introduced into FGSC33, FGSC237 and FGSC773, respectively, to generate *alcA*(p)::*vosA*, *vosA*(p)::VosA::RFP and *gpdA*(p)::RFP strains. The *vosA*(p)::VosA::FLAG construct was created by adding the FLAG sequence (DYKDDDDK) to the VosA C terminus. The *vosA*(p)::VosA::FLAG and *pyroA* ^+^ amplicons were co-introduced into RNI10.2 (*biA1*; *argB2*; *pyroA4*; Δ*vosA*::*argB* ^+^; *veA1*). To determine the *vosA* copy number, transformants showing the integration of VosA::FLAG in the genome were examined by real time PCR (qPCR) using SYBR® Premix *Ex Taq* (Takara) and ABI Prism 7900 (Perkin-Elmer/Applied Biosystems). The actin gene was used as a normalization control. A Δ*vosA* strain with a single copy *vosA*(p)::*vosA*::FLAG exhibiting the WT phenotype (shown in Fig 2A as a complemented strain) is used for Western blot analysis.

### Nucleic acid isolation and manipulation

Genomic DNA and total RNA isolation, and Northern blot analyses were carried out as described [35]. The DNA probes were prepared by PCR-amplification of the coding regions of individual genes with appropriate oligonucleotide pairs using FGSC4 genomic DNA as template (Table S2).

### Western blot

Sample preparation was done as described [44]. Protein concentrations were determined by a BCA protein assay kit (PIERCE). Proteins (20 μg/lane) were separated by electrophoresis in 7.5% SDS-polyacrylamide gel and electroblotted to the PVDF membrane (Bio-Rad). The VosA-FLAG protein was detected using mouse anti-FLAG M2 monoclonal antibody (Sigma), at a 1∶1,000 dilution and WesternBreeze® Chromogenic Kit–Anti-Mouse (Invitrogen). MagicMark™ XP Western Protein Size Standard (Invitrogen) was used.

### Spore viability test

Two-day old conidia of WT (FGSC26) and the mutant (RNI10.2) were spread on solid MM (10^5^/plate), incubated at 37°C, and the conidia from 2, 5, 10 and 20 day old cultures were collected. Approximately 250 conidia were inoculated on solid MM and incubated for 2∼3 days at 37°C until colonies appeared. Survival rate (triplicate/sample) was calculated as a ratio of the number of growing colonies to the number of spores inoculated.

### TEM

Conidia and ascospores were collected from the cultures on solid MM. Samples were fixed in Karnovsky's fixative (2% paraformaldehyde, 2.5% glutaraldehyde in 0.1 M Sorenson's sodium phosphate buffer [PB], pH 7.2) overnight at 4°C and post-fixed in 2% osmium tetroxide in PB for 1 h at RT. The samples were then dehydrated in a graded ethanol series from 35% to 100% and embedded in PolyBed812 resin (Polysciences). Polymerized samples were sectioned on a Leica UC6 ultramicrotome (80 nm) and stained with uranyl acetate and lead citrate. The stained sections were viewed on a JEOL 100CX transmission electron microscope, and documented with a SIS (Soft Imaging Systems, Lakewood, CO) MegaView III digital side mount camera.

### Trehalose assay and stress tolerance test

The amount of glucose liberated by the activity of trehalase was measured using a glucose assay kit (Sigma) and converted into the trehalose amount per conidium (23). Each sample not treated with trehalase served as a negative control. The experiments were performed in triplicate. To examine thermal tolerance, WT (FGSC26) and mutant (RNI10.2) conidia were incubated at 50°C for 0, 5, 20, 30, 45 or 60 min. To examine oxidative tolerance, WT or mutant conidia were treated with varying concentrations (0, 0.25, 0.5, 0.75 or 1 M) of H_2_O_2_ and incubated for 30 min at room temperature [45]. In both cases, the spores were inoculated on solid MM and incubated at 37°C for 48 h. Colony numbers were counted and calculated as a percentage of the untreated control.

### Yeast one-hybrid assay

The plasmids pGBDUC1, pNI32, pNI33, pNI34 and pNI35 were introduced into the *Saccharomyces cerevisiae* strain PJ69-4A (*MAT*a *trp1-901 leu2-3*, *112 ura3-52 his3-200 gal4*Δ *gal80*Δ *LYS2*::*GAL1*-*HIS3 GAL2*-*ADE2 met2*::*GAL7*-*lacZ*; [31]), respectively. Yeast media, transformation and drop test were carried out as described [46], [47]. Three transformants per plasmid were examined in the drop test. The LacZ activity was measured with the yeast β-galactosidase assay kit (Pierce).

### Microscopy

The colony photographs were taken using a Sony DSC-F828 digital camera. Photomicrographs were taken using an Olympus BH2 microscope equipped with the DP-70 digital imaging system. Fluorescence samples were prepared as described [48] and visualized by a Zeiss Axioplan 2 microscope with AxioVision digital imaging software (Zeiss).

## Supporting Information

Table S1 *Aspergillus* strains used in this study
(0.03 MB PDF)

Table S2 Oligonucleotides used in this study.
(0.05 MB DOC)

Figure S1 Overexpression of vosA is sufficient to direct the expression of trehalose biosynthetic genes in non-developing cells. Northern blot analyses for the levels of tpsA, tpsC and orlA mRNA in control (TJA53.1) and alcA(p)::vosA (TNI9.1) strains are shown. Two strains were grown in liquid glucose medium at 37°C, 250 rpm for 18 h and then transferred onto solid threonine medium (MMT; inducing) for the concomitant induction of conidiation and overexpression of vosA. It needs to be emphasized that such a synchronized induction of both conidiation and overexpression of vosA results in the absence of spore formation due to the prevailing inhibitory role of VosA, whereas a control strain produces a large number of conidia (for reference see Figure 4C). Thus, the mRNA levels of tpsA, tpsC and orlA in an alcA(p)::vosA strain represent those accumulate in undifferentiated hyphae, strongly supporting the role of VosA in activating the genes for trehalose biosynthesis.
(10.38 MB TIF)

Figure S2 Genetic interactions between vosA, brlA, abaA and wetA. Northern blot analyses for the levels of vosA, brlA, abaA and wetA transcripts in WT (FGSC26), brlA42 (AJC11.32), abaA14 (TTA021) and wetA6 (AJC1.22) strains are shown. brlA42, abaA14 and wetA6 are temperature sensitive alleles that exhibit loss of function at 37°C. The mutant conidia used for inoculation in liquid culture were collected from the colonies grown on solid MM at 28oC for 3 days. The strains were grown in liquid MM at 37°C, 250 rpm for 18 h (Veg 18 h) and then transferred onto solid MM and further incubated at 37°C. Samples were collected at designated time after transfer (Asexual 12, 24 and 48 h).
(4.16 MB TIF)

Figure S3 Effects of brlA and abaA overexpression on vosA, brlA, abaA and wetA. Northern blot analyses for the levels of vosA, brlA, abaA and wetA transcripts in WT (FGSC26), alcA(p)::brlA (OEbrlA, TTA292-1), alcA(p)::abaA (OEabaA, SJA7) and alcA(p)::brlA abaA14 (OEbrlA abaA14, TTA021) strains are shown. The strains were grown in liquid glucose medium (MMG) at 37°C, 250 rpm for 14 h and then transferred into liquid glucose medium (MMG) or liquid threonine medium (MMT; inducing). Note the high levels of brlA and abaA mRNA accumulation induced in liquid MMT. Samples were collected at designated time points after transfer.
(2.81 MB TIF)
