# Supplementary material for: A Novel Regulator Couples Sporogenesis and Trehalose Biogenesis in Aspergillus nidulans
Source: PLoS One. 2007 Oct 3;2(10):e970. doi: 10.1371/journal.pone.0000970 (PMC1978537; doi:10.1371/journal.pone.0000970)
Supplement: Table S1 — Aspergillus strains used in this study (0.03 MB PDF) [file pone.0000970.s001.pdf]

Table S1. *Aspergillus* strains used in this study

Strain	Genotype[a]	Source/Reference
A. nidulans		
FGSC4	*veA*$^+$	FGSC[b]
FGSC26	*biA1*	FGSC
FGSC33	*biA1*; *pyroA4*	FGSC
FGSC237	*pabaA1, yA2*; *trpC801*	FGSC
FGSC773	*pyrG89*; *wA3*; *pyroA4*	FGSC
PW1	*biA1*; *argB2*; *methG1*	P. Weglenski
RRAW16	*pyrG89*, *yA2*; *veA*$^+$	R. A. Wilson and N. P. Keller
JAS26	*argB2*; *pyroA4*	Seo and Yu, unpublished
TJA53.1	*pyrG89*; pJW53::*pyroA*$^+$	Seo and Yu, unpublished
RYG1.9	*pabaA1*, *yA2*; *argB2*; Δ*fluG*::*trpC*$^+$	Guan and Yu, unpublished
RNIW5	*pyrG89*; *pyroA4*	This study
TNI2.1	*argB2*; *pyroA4*; Δ*vosA*::*argB*$^+$	This study
RNI10.2	*biA1*; *argB2*; *pyroA4*; Δ*vosA*::*argB*$^+$	This study
RNI14.1	*biA1*; Δ*vosA*::*argB*$^+$; *veA*$^+$	This study
TNI10.34.1	*biA1*; *argB2*; *pyroA4*; Δ*vosA*::*argB*$^+$; *vosA*(p)::*vosA*::FLAG; *pyroA*$^+$	This study
TNI9.1, 2[C]	*biA1*; *alcA*(p)::*vosA*::*pyroA*$^+$	This study
TNI13. 1, 2, 3[C]	*pabaA1*, *yA2*; *vosA*(p)::*vosA*::RFP::*trpC*$^+$	This study
TNI20.1	*pyrG89*; *wA3*; *gpdA*(p)::RFP::*pyroA*$^+$	This study
AJC11.32	*biA1*; *brlA42*, *trpC399*	A. J. Clutterbuck
TTA021	*biA1*, *pabaA1*; *alcA*(p)::*brlA*; *abaA14*	1
AJC1.22	*biA1*; *wetA6*	A. J. Clutterbuck
TTA292-1	*biA1*; *argB*::*alcA*(p)::*brlA*; *methG1*	1
SJA7	*pabaA1*, *yA2*; *pyroA1*; *alcA*(p)::*abaA*	J. Aguirre
A. fumigatus		

AF293	WT	2
AF293.1	Af*pyrG1*	3
TNI17.1, 2, 3[C]	Af*pyrG1*; ΔAf*vosA*::Af*pyrG*$^+$	This study

---

[a]All *A. nidulans* strains carry the *veA1* mutation if not mentioned as *veA*$^+$.

[b]FGSC: Fungal Genetics Stock Center

[c]Multiple isogenic strains. They behaved identically.

1. Adams TH, Boylan MT, Timberlake WE (1988) *Cell* 54:353-362.

2. Brookman JL, Denning DW (2000) *Curr Opin Microbiol* 3:468-474.

3. Xue T, Nguyen CK, Romans A, Kontoyiannis DP, May GS (2004) *Arch Microbiol* 182:346-353.
