# Supplementary material for: A Novel Regulator Couples Sporogenesis and Trehalose Biogenesis in Aspergillus nidulans
Source: PLoS One. 2007 Oct 3;2(10):e970. doi: 10.1371/journal.pone.0000970 (PMC1978537; doi:10.1371/journal.pone.0000970)
Supplement: Table S2 — Oligonucleotides used in this study. (0.05 MB DOC) [file pone.0000970.s002.doc]

**Table S2 Oligonucleotides used in this study.**

--------------------------------------------------------------------------------------------------------------------------------------------

Oligo Sequence Position/Purpose

OMN33 aaa taa gct tgc atg cgc pRG3-AMA1 sequencing 5′

OMN35 gcc agt gaa ttc gag ctc pRG3-AMA1 sequencing 3′

OMN148 aat ccc aag tcc aac cgt gag Forward primer for actin for qPCR

OMN149 aag atc ttc atc agg tag tcc g Reverse primer for actin for qPCR

OMN150 gta gac aac aca ggt aaa gag c Forward primer for *vosA* for qPCR

OMN151 tgg att tca gaa aca caa cgt c Reverse primer for *vosA* for qPCR

OKH13 cag ttg agc ctg aga cca at Forward primer for *pyroA*^+^

OKH14 cgc ttt ctc ttg gct cta tc Reverse primer for *pyroA*^+^

OKH60 gac tct ata cca ccg tac gcc gat at Forward primer for *argB*^+^

OKH67 gtg tta ggc ctg gat cta Reverse primer for *argB*^+^

OKH235 gcc tca aac aat gct ctt cac cct Forward primer for Af*pyrG*^+^

OKH236 att ctg tct gag agg agg cac tga Reverse primer for Af*pyrG*^+^

OMN54 ttt ttg ccg ctg ctg gag tta g  *vosA* deletion 5′ forward

OMN55 aag agg gct ttg tgg ggt ttt c *vosA* deletion 3′ reverse

OMN56 ***caa atg agg cct cta aac tgg tc a*** *vosA* deletion 5′ reverse with complementary

gag cac tat gag aga cga ctg 5′ *argB*^+^ tail (bold italic)

OMN57 ***caa ggt aga tcc agg cct aac ac a***  *vosA* deletion 3′ forward with complementary

gga ttc tcg ttt gtg gaa cac 3′ *argB*^+^ tail (bold italic)

OMN58 gct ata aca aag aga gag agg g *vosA* deletion construct 5′ nest

OMN61 gcg taa act caa cag ctg aag c  *vosA* deletion construct 3′ nest

OMN107 g***ga att c***ct ggc cat cta cat cgt c Af*vosA* deletion 5′ forward with *Eco*RI (bold italic)

OMN108 g***ga att c***cg gaa tat cag ctt acc tca a Af*vosA* deletion 3′ reverse with *Eco*RI (bold italic)

OMN109 ***g gtg aag agc att gtt tga ggc a*** Af*vosA* deletion 5′ reverse with complementary

ggc gaa gat ctt gtc aaa gaa g 5′ *pyrG*^+^ tail (bold italic)

OMN110 ***agt gcc tcc tct cag aca gaa t a*** Af*vosA* deletion 3′ forward with complementary

a ccc cga taa tgg att tac gcg 3′ *pyrG*^+^ tail (bold italic)

OMN111 tca tct acg aac tgc tgc ctt g Af*vosA* deletion construct 5′ nest

OMN112 caa gct cat tgc cat tcc aag t Af*vosA* deletion construct 3′ nest

OJA141 ccg ttc tgc tta ggg ta 5′ end of *alcA*(p)

OJA106 ttt gag gcg agg tga tag gat tgg a 3′ end of *alcA*(p)

OJA108 cg ***gga tcc*** agt ggt tcg gta atc *alcA*(p) 5′ nested with *Bam*HI tail (bold italic)

OMN70 ***tcc aat cct atc acc tcg cct caa a*** *vosA* overexpression 5′ with complementary

agt gct cgc ctt gat gag tg *alcA*(p) tail (bold italic)

OMN88 g **gaattc** cga ***agatct*** cca ggt tgt cat ac *vosA* overexpression construct 3′ nested with

*Eco*RI (bold) and *Bgl*II (bold italic)

OMN101 cg **ggatcc** ga ***agatct*** tca gac cca tcg ttc agc *vosA*(p)::*vosA* forward with *Bam*HI (bold)

and *Bgl*II (bold italic)

OMN106 cg **ggatcc** ga ***agatct*** tca aca cag agc ttg cct ct *vosA*(p)::*vosA* reverse with *Bam*HI (bold)

and *Bgl*II tail (bold italic)

OMN114 gac tct gcc gac agg aaa aca g *vosA* promoter 5′ forward

OMN116 cgg agg agg cca t**tg gac c** rev 5' RFP + Gly Pro (bold)+ rev *vosA* 3' end w/o STOP

ccg agg agt tcc gtt cgc tga g

OMN117 **ggt cca** atg gcc tcc tcc gag gac gtc Gly Pro (bold) + RFP amplification 5' forward

OMN118 g a***ag atc t***tc gaa ttc tta ggc gcc ggt g RFP 3′ reverse with *Bgl*II tail (bold italic)

OJA225 ggt tat gct agt tat tgc tca RFP amplification 3′ Reverse

OMN81 ttg cta gca gtt cca ggt gg *gpdA*(p) 5′ amplification

OMN80 tgt gat gtc tgc tca agc gg *gpdA*(p) 3′ amplification

OMN120 g ***gaa ttc*** ggc acg gct acg gaa gac  *gpdA*(p) 5' nested with *Eco*RI (bold italic)

OMN121 ***cc gct tga gca gac atc aca*** RFP 5' amplification with *gpdA*(p) 3′ tail (bold italic)

tga cga taa gga tcc ga gg

OJA223 tc***a agc tt***c **gaa ttc** tta ggc RFP 3′ reverse containing *Eco*RI (bold) and *Hin*dIII (bold italic)

OMN122 ga **agatct** tca ***ctt gtc gtc gtc gtc ttt gta gtc***  *Bgl*II (bold underlined) + stop + rev FLAG

ccg agg agt tcc gtt cgc tg (DYKDDDDK) (bold italic) + 3' *vosA* ORF

OMN145 ***ac gac gac aag*** tga **agatct** tc gga ttc tcg ttt part of FLAG (bold italic) + stop + *Bgl*II

gtg gaa cac c (bold) + tc + *vosA* 3’ UTR

OMN184 cg ***ggatcc*** atg agt gcg gcg aac tat cca g VosA N-terminal 5′ end with *Bam*HI tail (bold italic)

OMN185 cg ***ggatcc*** atg caa atc cct gga agt agc VosA C-terminal 5′ end with *Bam*HI tail (bold italic)

OMN186 cc ***atcgat*** ggc tgt cct atc aga ggg aga g VosA N-terminal 3′ end with *Cla*I tail (bold italic)

OMN190 cc ***atcgat*** tcg agg agt tcc gtt cgc tga g VosA C-terminal 3′ end with *Cla*I tail (bold italic)

OMN228 g ***gaattc*** atg gag ccc cca gcg atc ag AflR 5′ end with *Eco*RI tail (bold italic)

OMN229 cg ***ggatcc*** ggc gtg gcg gag gat gct gat c AflR 3′ end with *Bam*HI tail (bold italic)

OMN66 ttt cca gat cct tcg cag *vosA* forward primer for probe

OMN63 ata gaa aca gcc acc cag *vosA* reverse primer for probe and RT-PCR

OMN164 ggt gtc aaa cta cgc atc ag Af*vosA* forward primer for probe

OMN165 cat tat gtg agc cag gat gc Af*vosA* reverse primer for probe

OJA142 ctg gca ggt gaa caa gtc *brlA* forward primer for probe

OJA143 aga agt taa cac cgt aga *brlA* reverse primer for probe

OJA154 agc tct tca gaa tac gtc *abaA* forward primer for probe

OJA155 gtt gtg ata tgc ctc cat *abaA* reverse primer for probe

OJA150 cag tac gtc aat atg gac *wetA* forward primer for probe

OJA151 gtg aag ttg aca aac gac *wetA* reverse primer for probe

OJA156 atg cta tat tca cca cct *rodA* forward primer for probe

OJA157 tga cct acc aga ata tcg *rodA* reverse primer for probe

OMN128 gac cgt gag tct gag tat tc *wA* forward primer for probe

OMN129 tga atc act gta ata ccg cc *wA* reverse primer for probe

OJA152 aac tct cga gct gac atg *yA* forward primer for probe

OJA153 ctt gga cat tat gta ggt *yA* reverse primer for probe

OMN176 cca tca cca taa agc gat cag *tpsA* forward primer for probe

OMN177 cag ttt cga gaa gtt aag cgc *tpsA* reverse primer for probe

OMN178 cga gta cgc caa tca att ccg *treA* forward primer for probe

OMN179 agt aga ggc aca ggc tgg atc *treA* reverse primer for probe

OMN180 aag att ctc aac gcc aac cgg *treB* forward primer for probe

OMN181 cgg gtc aca tcg tat ttc tcg *treB* reverse primer for probe

OMN182 cag ccg cat ctc caa ctt ag  *orlA* forward primer for probe

OMN183 tgt tag cag caa ttc atc gcg *orlA* reverse primer for probe

OMN319 tac gag tcc acg ctc tcc agt g  *tpsC* forward primer for probe

OMN320 gga gca tac ctt gta ttg ttg gt *tpsC* reverse primer for probe

--------------------------------------------------------------------------------------------------------------------------------------------
