# Supplementary figures and images for: A Novel Regulator Couples Sporogenesis and Trehalose Biogenesis in Aspergillus nidulans
Source: PLoS One. 2007 Oct 3;2(10):e970. doi: 10.1371/journal.pone.0000970 (PMC1978537; doi:10.1371/journal.pone.0000970)

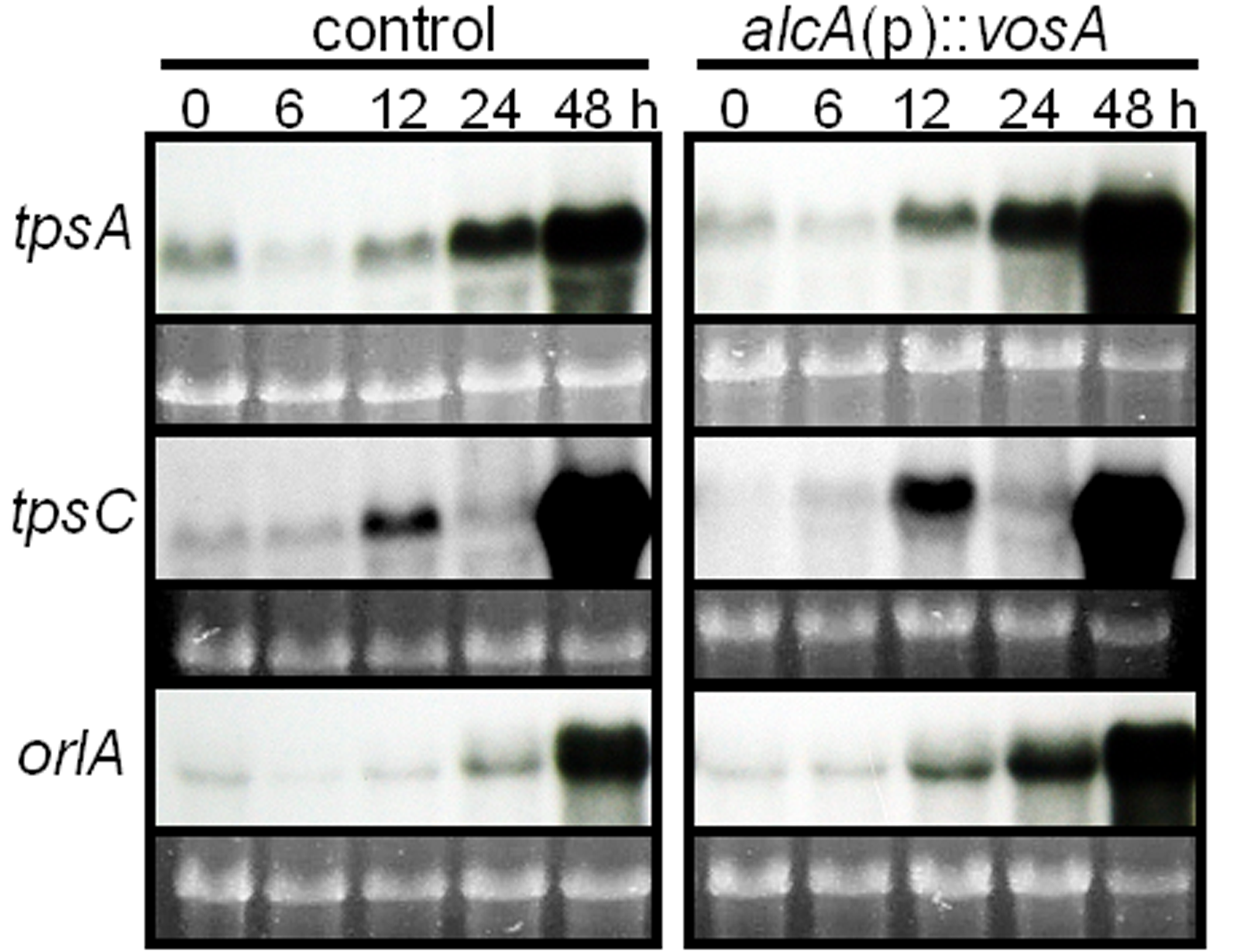

Supplement: Figure S1 — Overexpression of vosA is sufficient to direct the expression of trehalose biosynthetic genes in non-developing cells. Northern blot analyses for the levels of tpsA, tpsC and orlA mRNA in control (TJA53.1) and alcA(p)::vosA (TNI9.1) strains are shown. Two strains were grown in liquid glucose medium at 37°C, 250 rpm for 18 h and then transferred onto solid threonine medium (MMT; inducing) for the concomitant induction of conidiation and overexpression of vosA. It needs to be emphasized that such a synchronized induction of both conidiation and overexpression of vosA results in the absence of spore formation due to the prevailing inhibitory role of VosA, whereas a control strain produces a large number of conidia (for reference see Figure 4C). Thus, the mRNA levels of tpsA, tpsC and orlA in an alcA(p)::vosA strain represent those accumulate in undifferentiated hyphae, strongly supporting the role of VosA in activating the genes for trehalose biosynthesis. (10.38 MB TIF) [file pone.0000970.s003.tif]

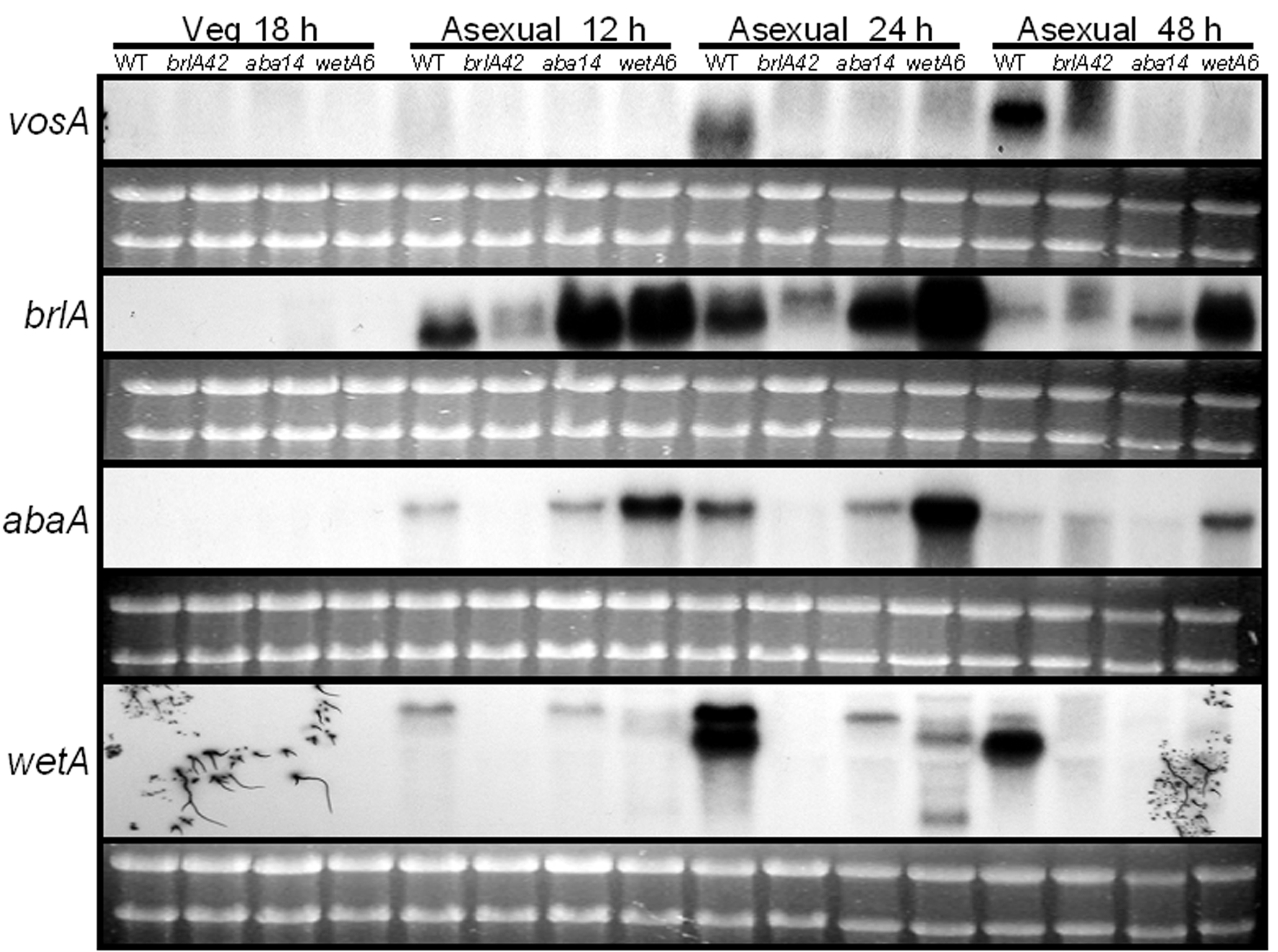

Supplement: Figure S2 — Genetic interactions between vosA, brlA, abaA and wetA. Northern blot analyses for the levels of vosA, brlA, abaA and wetA transcripts in WT (FGSC26), brlA42 (AJC11.32), abaA14 (TTA021) and wetA6 (AJC1.22) strains are shown. brlA42, abaA14 and wetA6 are temperature sensitive alleles that exhibit loss of function at 37°C. The mutant conidia used for inoculation in liquid culture were collected from the colonies grown on solid MM at 28oC for 3 days. The strains were grown in liquid MM at 37°C, 250 rpm for 18 h (Veg 18 h) and then transferred onto solid MM and further incubated at 37°C. Samples were collected at designated time after transfer (Asexual 12, 24 and 48 h). (4.16 MB TIF) [file pone.0000970.s004.tif]

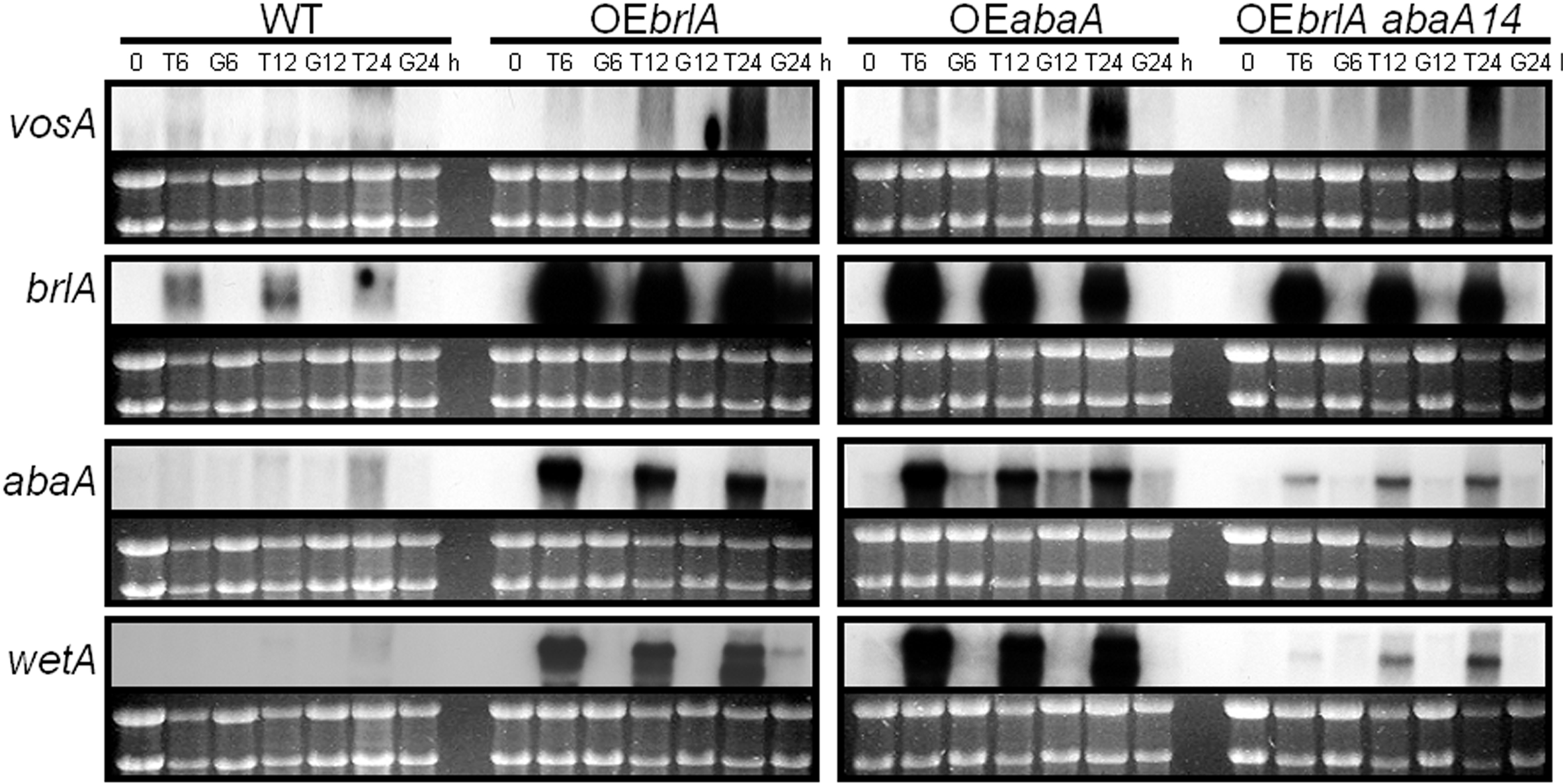

Supplement: Figure S3 — Effects of brlA and abaA overexpression on vosA, brlA, abaA and wetA. Northern blot analyses for the levels of vosA, brlA, abaA and wetA transcripts in WT (FGSC26), alcA(p)::brlA (OEbrlA, TTA292-1), alcA(p)::abaA (OEabaA, SJA7) and alcA(p)::brlA abaA14 (OEbrlA abaA14, TTA021) strains are shown. The strains were grown in liquid glucose medium (MMG) at 37°C, 250 rpm for 14 h and then transferred into liquid glucose medium (MMG) or liquid threonine medium (MMT; inducing). Note the high levels of brlA and abaA mRNA accumulation induced in liquid MMT. Samples were collected at designated time points after transfer. (2.81 MB TIF) [file pone.0000970.s005.tif]
